# Gaps in immunization coverage at school entry and after two years of school attendance among immigrant and refugee children in Ontario, Canada

**DOI:** 10.1371/journal.pone.0330955

**Published:** 2025-09-09

**Authors:** Sarah E. Wilson, Catharine Chambers, Andrew S. Wilton, Li Bai, Natasha Crowcroft, Shelley L. Deeks, Scott A. Halperin, Jeffrey C. Kwong, Meb Rashid, Karen Tu, Kumanan Wilson, Astrid Guttmann

**Affiliations:** 1 Public Health Ontario, Toronto, Ontario, Canada; 2 Dalla Lana School of Public Health, University of Toronto, Toronto, Ontario, Canada; 3 ICES, Toronto, Ontario, Canada; 4 Community Health and Epidemiology, Dalhousie University, Nova Scotia, Canada; 5 Department of Health and Wellness, Government of Nova Scotia, Halifax, Nova Scotia, Canada; 6 Canadian Center for Vaccinology & IWK Health Center, Halifax, Nova Scotia, Canada; 7 Department of Pediatrics, Faculty of Medicine, Dalhousie University, Halifax, Nova Scotia, Canada; 8 Department of Family and Community Medicine, Temerty Faculty of Medicine, University of Toronto, Toronto, Ontario, Canada; 9 Toronto Western Family Health Team, University Health Network, Toronto, Ontario, Canada; 10 Crossroads Clinic, Women’s College Hospital, University of Toronto, Toronto, Ontario, Canada; 11 North York General Hospital, Toronto, Ontario, Canada; 12 Institute for Health Policy, Management, and Evaluation, University of Toronto, Toronto, Ontario, Canada; 13 Department of Medicine, University of Ottawa, Ottawa, Ontario, Canada; 14 Bruyere Health Research Institute, Ottawa, Ontario, Canada; 15 Ottawa Hospital Research Institute, Ottawa, Ontario, Canada; 16 Department of Paediatrics, The Hospital for Sick Children, Toronto, Ontario, Canada; 17 Edwin S. H. Leong Centre for Healthy Children, University of Toronto, Toronto, Ontario, Canada; University of Illinois Urbana-Champaign College of Veterinary Medicine, UNITED STATES OF AMERICA

## Abstract

**Background:**

Foreign-born children may face greater barriers to accessing routine immunizations in Canada or their country of birth, but provincial surveillance data on immigration status are lacking. Using our provincial immunization repository linked to administrative data, we assessed immunization coverage among immigrant and refugee children in Ontario, Canada, compared with Ontario-born children and identified factors associated with being up-to-date (UTD).

**Methods:**

We conducted a retrospective cohort study of children entering school during the 2012/13–2014/15 school years. We calculated UTD coverage for measles (2 doses), diphtheria (4 doses), and polio (3 doses) vaccines at school entry and two years after school attendance. We compared UTD coverage between immigrant/refugee children and Ontario-born children using standardized differences (SD).

**Results:**

In a cohort of 363,662 children, 15,114 (4.2%) were immigrants/refugees (82.1% immigrants, 17.9% refugees). UTD coverage for all antigens combined was 59.2% among immigrant/refugee children compared with 87.9% among Ontario-born children at school entry (SD = 0.69), increasing to 84.9% and 94.3%, respectively, two years after school entry (SD = 0.31). Coverage was lower with greater disparities between immigrant/refugee and Ontario-born children for measles (87.9% vs. 94.8%, SD = 0.25) and diphtheria (94.6% vs. 97.4%, SD = 0.15) after two years than polio (97.1% vs. 98.4%, SD = 0.09). Among immigrant/refugee children, coverage was lowest in refugees (vs. immigrants), recent immigrants, and those born in certain regions.

**Conclusions:**

Immunization coverage among foreign-born children lagged behind their Ontario-born peers, even after two years of school attendance. Findings varied by vaccine, immigration category, time spent in Ontario, and country of birth.

## Introduction

The proportion of immigrants in Canada is increasing and is now the main driver of Canada’s population growth [1]. About one in every six newcomers to Canada are children [1]. Immigrants and refugees may be more susceptible to vaccine-preventable diseases (VPDs) and their associated morbidity and mortality due to inconsistent access to health services, including immunizations [2–4]. Compared with Canadian-born individuals, newcomers to Canada may have lacked access to comprehensive publicly funded immunization programs or had different immunization schedules or fewer vaccines available in their country of birth, resulting in lower coverage upon arrival [5]. They may also face greater barriers to accessing healthcare within Canada, including language or cultural barriers, socio-economic constraints, unfamiliarity with the Canadian healthcare system or immunization schedules, mistrust of the healthcare system, misinformation or lack of information about vaccines, and/or systemic racism [6–8]. Past studies in Ontario, Canada, among children born in Canada to immigrant and refugee mothers have shown that immunization uptake may in some instances be higher than among children of non-immigrants [9–11]. However, outside of what is known for COVID-19 vaccine [12], there is very limited information on immunization trends among school-aged immigrant and refugee children themselves.

Ontario is Canada’s most populous and ethnically diverse province with 14.2 million residents. Approximately 30% of Ontario residents are immigrants relative to 23% for the rest of Canada [13], which itself is the highest among G7 countries [1]. Under Ontario’s routine publicly funded immunization schedule, children starting immunization in infancy are eligible to receive four doses of the diphtheria, tetanus, acellular pertussis, inactivated poliovirus, and *Haemophilus influenzae* type B (DTaP-IPV-Hib) vaccine at 2, 4, 6, and 18 months of age and one dose of the measles, mumps, and rubella (MMR) vaccine at 1 year of age [14]. Additionally, children receive a single pre-school booster dose of the tetanus, diphtheria, acellular pertussis, and inactivated poliovirus (Tdap-IPV) vaccine and measles, mumps, rubella, and varicella (MMRV) vaccine between 4 and 6 years of age. (Prior to 2011, the second dose of MMR was given at 18 months of age in Ontario, while prior to 2015, DTaP-IPV was used for the pre-school booster instead of Tdap-IPV.) Children who missed doses, for example immigrant and refugee children arriving in Canada after the scheduled ages, are eligible for catch-up immunization according to various schedules, depending on age and prior immunization history [14].

As part of its National Immunization Strategy, the Public Health Agency of Canada has set vaccination coverage goals to reach herd immunity thresholds, aiming to achieve 95% coverage for four doses of DTaP, three doses of polio-containing vaccine, and one dose of MMR among 2-year-olds by 2025 [15]. Immunization coverage in Ontario currently falls short of these goals, even prior to the COVID-19 pandemic [16]. Uptake is anticipated to be even lower in specific populations, such as immigrant or refugee children [3,17–23]; however, Ontario’s provincial immunization repository does not capture information on country of birth, precluding our ability to measure these gaps in coverage and identify subgroups at potentially higher risk for VPDs. Through linkage to health and demographic administrative data, our objectives were to assess up-to-date (UTD) immunization coverage among immigrant and refugee children in Ontario at the time of school entry at ages 5 and 6 and then two years later compared with Ontario-born students at the same time points and to identify factors associated with being UTD for childhood immunizations. We focused our analysis on coverage of measles, diphtheria, and polio to represent antigens contained in the DTaP/Tdap, IPV, and MMR vaccines.

## Methods

### Study population and setting

All Ontario residents have access to publicly funded, routine medical services, including immunizations, through the single-payer Ontario Health Insurance Plan (OHIP). During the study period, immigrants admitted to Canada as permanent residents (i.e., those who have been granted the right to stay and work in Canada without limitations on their stay) and refugees (including those granted emergency authorization to temporarily live in Canada for humanitarian reasons) were eligible for OHIP after 3 months of residence [24]. Despite publicly funded immunization services, Ontario does not have a comprehensive real-time immunization registry [25]. Instead, parents or guardians are required to report their child’s immunizations, which are typically administered in primary care physician’s offices, into the provincial Digital Health Immunization Repository (DHIR) at the time of school enrollment. Parents can do this electronically through a reporting portal, or they can send their child’s immunization history to the public health unit for subsequent data entry. Data completeness for the DHIR is assumed to be high during the study period, with sensitivity exceeding 95% across vaccines compared with electronic medical records from a primary care network when assessed using a cohort of Ontario-born children [26]. Separate from the process of parental/guardian reporting, schools or school boards upload enrollment lists into the DHIR to establish a population-based roster for all students in Ontario. Local Public Health Units assess student’s immunization status and notify parents or guardians if children are overdue for select immunizations outlined in Ontario’s school entry immunization legislation [27]. Students who have not provided valid immunization information or a statement of medical or non-medical (i.e., religious or conscientious) exemption may face school suspension.

### Study design and cohort creation

**Fig 1:**
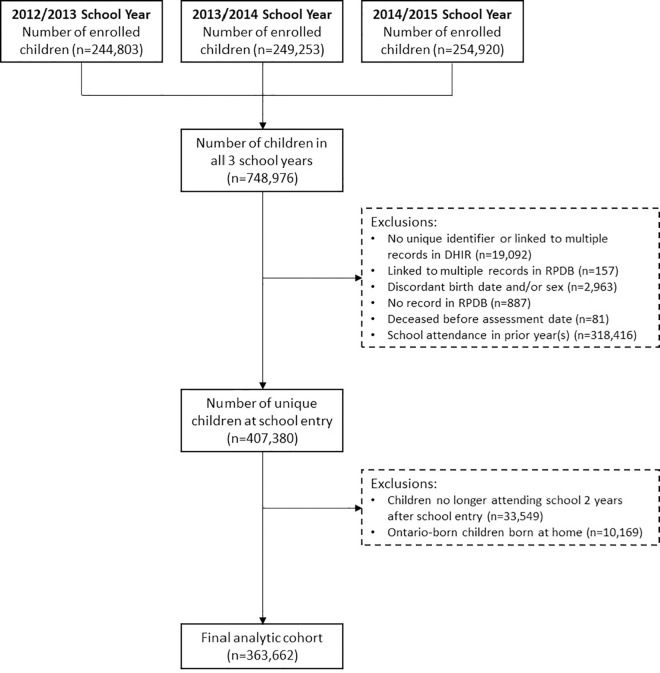
Cohort creation flowchart.

We conducted a retrospective, population-based cohort study. We identified children entering school in kindergarten or grade 1 (ages 5 or 6) during the 2012/13–2014/15 school years, representing the 2006–2009 birth cohorts, who had evidence of new school attendance in the relevant school year using client records from the DHIR previously linked to the provincial health insurance registry (Registered Persons Database [RPDB]), enabling linkage to a variety of health and demographic datasets used for this study. We restricted the cohort to children who had evidence of school attendance in Ontario for at least two years after entry. We excluded children with no unique identifier or those linked to multiple records in the DHIR or RPDB, children with discordant birth dates and/or sex, and children without a record in the RPDB (Fig 1). We also excluded Ontario-born children born at home (vs. in hospital), as attitudes and beliefs towards immunizations may differ among individuals who choose to have a midwife-assisted home birth [28]. These datasets were linked using unique encoded identifiers and analyzed at ICES, an independent, non-profit research institute in Ontario [29]. The data were accessed for research purposes on 02/10/2020.

### Covariates and additional data sources

Our primary exposure variable was immigrant or refugee status. We used the Immigration, Refugee, and Citizenship Canada (IRCC) Permanent Resident Database, a federal database consisting of socio-demographic and immigration information on all permanent residents and refugees who landed in Ontario from 1985 to May [31], 2017, to determine if the child was a landed immigrant or refugee. Overall probabilistic linkage of the IRCC data to the RPDB identifies about 86% of immigrants [30]. Among immigrant/refugee children, we described the visa type, time since landing date, region of birth based on modified World Bank Regions [31], and identified the top 10 countries of birth. We identified children born in Ontario hospitals using the MOMBABY database, which is comprised of hospital admission records of birthing parents and their newborns. Children without a record in MOMBABY but who became eligible for OHIP within the first week of life (i.e., home births) were excluded. We defined a third group of children born outside of Ontario as those without a record in the IRCC Permanent Resident Database or MOMBABY. This group is comprised of children born in other Canadian provinces/territories, immigrant and refugee children who landed in a different province/territory, and undocumented immigrant and refugee children.

We used the DHIR to identify the child’s sex, birth year, school type, and postal code. Postal code was linked to the 2011 Census to determine rural residence (community size < 10,000) and Dissemination Area (DA), the smallest standard geographic unit available, using the Postal Code Conversion File. We used the 2011 Ontario Marginalization Index (ON-Marg) to assign DAs to one of five quintiles of material resources (previously described as material deprivation) [32]. We determined the model of primary care delivery from birth to 36 months using the Client Agency Patient Enrollment database, OHIP billing codes for primary care visits, and Community Health Centre visits [12].

### Analysis

We calculated UTD immunization coverage for select VPDs (measles, diphtheria, polio, and a composite measure for all three antigens) using the DHIR. We defined UTD coverage consistent with provincial surveillance definitions used at Public Health Ontario for the number of doses expected by the age of the children included in the study [33]. For diphtheria, this included receipt of 4 valid doses (primary series and toddler booster); for polio, this included 3 valid doses (primary series only); and for measles, this included 2 valid doses. For measles, we used a 2-dose UTD definition following guidance from Canada’s National Advisory Committee on Immunization (NACI), which states that the second dose should be administered before school entry [34]; all children in these cohorts would have been eligible for their second MMR dose at 18 months of age. UTD coverage was calculated at the start of the school year at the time of school entry (vaccine administration dates on or before September 30^th^ of the relevant year, 2012/13–2014/15) and at the end of the school year two years after school entry (administration dates on or before June 30^th^ of the relevant year, 2014/15–2016/17). We compared UTD coverage across groups of children based on their place of birth using standardized differences (SD), with Ontario-born children as the reference group; SD > 0.1 were considered to be meaningful differences [35]. SDs are not as sensitive to sample size as traditional statistical significance tests [36]. All analyses were conducted in SAS version 9.4 (SAS Institute, Cary, NC).

### Ethics review

This study was approved by the ethics review board of Public Health Ontario. ICES is a prescribed entity under Ontario’s Personal Health Information Protection Act (PHIPA). Section 45 of PHIPA authorizes ICES to collect personal health information, without consent, for the purpose of analysis or compiling statistical information with respect to the management of, evaluation or monitoring of, the allocation of resources to or planning for all or part of the health system. Thus, the ethics review of board of Public Health Ontario waived the requirement for informed consent for this study.

## Results

### Descriptive characteristics

Approximately 250,000 children 5–6 years old were enrolled in Ontario schools each year during the 2012/13–2014/15 school years. After removing duplicate records for children enrolled in more than one school year (retaining the year of school entry), children who were no longer attending school after two years, and exclusions related to data quality, the final analytic cohort consisted of 363,662 children (Fig 1). More than 90% of the cohort (n = 330,628) were born in an Ontario hospital, while 4.2% (n = 15,114) were immigrant or refugee children and 4.9% (n = 17,920) were born outside of Ontario (Table 1). Most Ontario-born children received primary care from family medicine teams (73.4%) or pediatricians (19.6%) from birth to 36 months of age, while most immigrant/refugee children (73.6%) received primary care in the first three years of life outside the province, as they arrived in Ontario after 36 months of age. Compared with Ontario-born children, immigrant/refugee children were more likely to live in urban areas or neighbourhoods with low material resources.

**Table 1:** Demographic characteristics of children at school entry in Ontario, Canada, 2012/13 to 2014/15 school years.

Characteristic	Ontario-born children (n = 330,628)	Immigrant or refugee children (n = 15,114)	Children born outside of Ontario (n = 17,920)*
	n (%)	n (%)	n (%)
School year			
2012/13	105,639 (32.0)	5,213 (34.5)	5,742 (32.0)
2013/14	108,841 (32.9)	4,838 (32.0)	5,809 (32.4)
2014/15	116,148 (35.1)	5,063 (33.5)	6,369 (35.5)
Female sex	161,582 (48.9)	7,254 (48.0)	8,692 (48.5)
Urban residence	296,667 (89.7)	14,985 (99.1)	16,594 (92.6)
Primary care group (birth to 36 months)			
Community Health Centre	2,813 (0.9)	51 (0.3)	55 (0.3)
Family medicine†	242,613 (73.4)	2,177 (14.4)	4,503 (25.1)
Pediatrician	64,803 (19.6)	313 (2.1)	814 (4.5)
Other^‡^	18,081 (5.5)	505 (3.3)	680 (3.8)
Lived outside of Ontario	—	11,122 (73.6)	10,498 (58.6)
No primary care visits	2,318 (0.7)	946 (6.3)	1,370 (7.6)
School board type			
Public	317,947 (96.2)	14,736 (97.5)	16,923 (94.4)
Private or other	12,681 (3.8)	378 (2.5)	997 (5.6)
Material resources quintile			
Q1 (highest)	65,525 (19.8)	1,180 (7.8)	3,698 (20.6)
Q2	69,313 (21.0)	1,602 (10.6)	3,327 (18.6)
Q3	63,842 (19.3)	2,141 (14.2)	3,042 (17.0)
Q4	60,726 (18.4)	3,370 (22.3)	3,179 (17.7)
Q5 (lowest)	69,758 (21.1)	6,804 (45.0)	4,570 (25.5)
Missing	1,464 (0.4)	17 (0.1)	104 (0.6)
Immigration category			
Immigrant	—	12,414 (82.1)	—
Economic-class		10,030 (66.4)	
Family-sponsored		2,384 (15.8)	
Refugee	—	2,700 (17.9)	—
Government-assisted		555 (3.7)	
Privately-sponsored^§^		503 (3.3)	
Asylum seekers		777 (5.1)	
Other**		865 (5.7)	
Time since arriving in Ontario			
< 1 year	—	4,252 (28.1)	—
1–2 years	—	5,524 (36.6)	—
3–4 years	—	4,939 (32.7)	—
≥ 5 years	—	399 (2.6)	—
Region of birth			
USA/UK/Western Europe	—	2,560 (16.9)	—
Central America/Caribbean	—	582 (3.9)	—
South America	—	344 (2.3)	—
Eastern Europe/Central Asia	—	893 (5.9)	—
Sub-Saharan Africa	—	1,029 (6.8)	—
Middle East/North Africa	—	3,001 (19.9)	—
East Asia and Pacific	—	2,171 (14.4)	—
South Asia	—	4,534 (30.0)	—
Top 10 countries of birth			
India	—	2,342 (15.5)	—
USA	—	1,550 (10.3)	—
Pakistan	—	1,145 (7.6)	—
China, People’s Republic of	—	862 (5.7)	—
Philippines	—	768 (5.1)	—
United Arab Emirates	—	701 (4.6)	—
Bangladesh	—	500 (3.3)	—
Iraq	—	386 (2.6)	—
Egypt	—	353 (2.3)	—
Nigeria	—	338 (2.2)	—

UK=United Kingdom; USA=United States of America.

*Includes children born in other Canadian provinces/territories, immigrant and refugee children who landed in a different province/territory, and undocumented immigrant and refugee children.

^†^Includes general practitioners, community medicine specialists, pediatricians and nurse practitioners who provide primary care in a family health team or through a comprehensive care model.

^‡^Includes walk-in clinics, solo-practitioners, and those with no primary care model.

^§^Includes blended visa office-referred refugees.

**Includes refugee dependents, refugees admitted on humanitarian and compassionate grounds, and public policy consideration cases.

Among immigrant/refugee children, most were economic-class (66.4%) or family-sponsored (15.8%) immigrants, while 17.9% were refugees (Table 1). About two-thirds had been in Ontario for less than three years, with 28.1% having a landing date within the year prior to school enrollment. Most were born in South Asia (30.0%), the Middle East or North Africa (19.9%), or the United States, the United Kingdom, or Western Europe (16.9%); almost half of all immigrant/refugee children were born in just six countries (India, United States, Pakistan, China, Philippines, and United Arab Emirates).

### Immunization coverage at school entry and two years later

**Fig 2:**
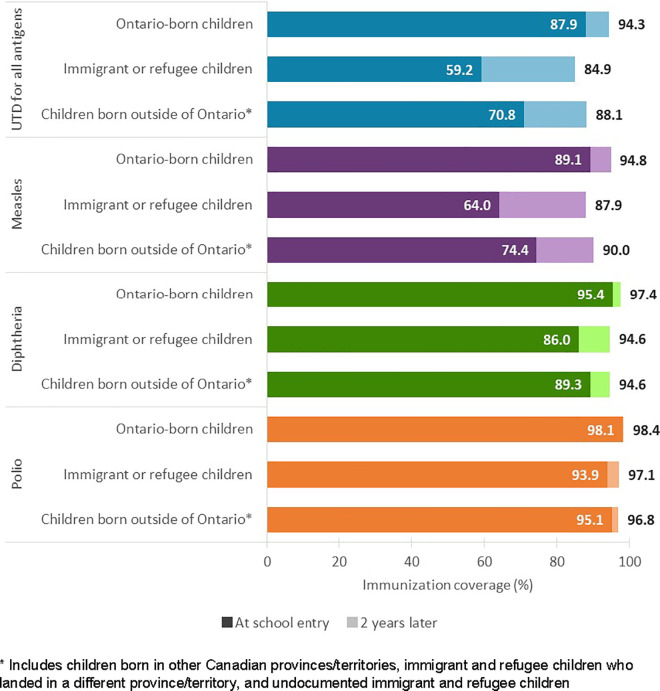
Up-to-date immunization coverage for measles, diphtheria, and polio assessed at school entry and two years after school entry among children in Ontario, Canada, 2012/13 to 2016/17 school years.

At school entry, UTD immunization coverage for all antigens combined was 59.2% among immigrant/refugee children compared with 87.9% for Ontario-born children and 70.8% for children born outside of Ontario (Fig 2). Two years later, immigrant/refugee children had the largest gains in immunization coverage, increasing to 84.9% for all antigens (change=+25.7%), but still lagged compared with Ontario-born children (94.3%, change=+6.4%) and children born outside of Ontario (88.1%, change=+17.3%). We observed similar patterns for individual antigens, with measles having the lowest coverage both at school entry (64.0% among immigrant/refugee children vs. 89.1% among Ontario-born children, SD = 0.62) and two years later (87.9% among immigrant/refugee children vs. 94.8% among Ontario-born children, SD = 0.25). We found higher coverage, meeting or exceeding 95% after two years of school attendance in all groups of children, for diphtheria and polio.

### Immunization coverage by covariates

Two years after school entry, UTD immunization coverage was lower in immigrant/refugee children compared with Ontario-born children across all covariates (SD > 0.10), with the exception of children receiving care from pediatricians and ‘other’ primary care models where coverage was similar and high (exceeding 94% and 90%, respectively) and children with no regular primary care where coverage was similar and low (below 85%) (Table 2). These differences were most pronounced for measles, with coverage for diphtheria and polio being more similar between immigrant/refugee children and Ontario-born children when assessed after two years.

**Table 2:** Up-to-date immunization coverage for measles, diphtheria, and polio assessed at two years after school entry among immigrant and refugee children compared with children born in Ontario, Canada, 2014/15 to 2016/17 school years.

Characteristic	UTD coverage (%)											
	UTD for all antigens			Measles			Diphtheria			Polio		
	Ontario-born children	Immigrant or refugee children	SD^*^	Ontario-born children	Immigrant or refugee children	SD^*^	Ontario-born children	Immigrant or refugee children	SD^*^	Ontario-born children	Immigrant or refugee children	SD*
Overall	94.3	84.9	0.31	94.8	87.9	0.25	97.4	94.6	0.15	98.4	97.1	0.09
School year at entry												
2012/13	92.9	78.9	0.41	93.5	82.5	0.34	97.2	92.9	0.20	98.4	96.3	0.13
2013/14	95.5	88.7	0.26	96.0	91.3	0.19	97.7	95.6	0.11	98.5	97.8	0.05
2014/15	94.6	87.4	0.25	95.0	90.2	0.18	97.4	95.3	0.12	98.3	97.4	0.06
Sex												
Female	94.4	84.6	0.33	94.9	87.8	0.26	97.5	94.3	0.16	98.4	96.8	0.11
Male	94.3	85.2	0.30	94.7	88.0	0.24	97.4	94.8	0.13	98.4	97.4	0.07
Urban residence												
Yes	94.3	84.8	0.31	94.7	87.9	0.25	97.5	94.6	0.15	98.5	97.1	0.09
No	94.9	90.3	0.18	95.5	91.9	0.15	96.6	94.4	0.11	97.7	96.8	0.06
Primary care group (birth to 36 months)												
Community Health Centre	95.6	80.4	0.48	96.1	84.3	0.40	97.9	96.1	0.11	98.7	98.0	0.05
Family medicine^†^	94.4	89.8	0.17	94.9	91.9	0.12	97.4	95.9	0.08	98.4	98.1	0.02
Pediatrician	95.1	93.9	0.05	95.3	95.9	0.03	98.3	96.8	0.10	99.1	98.7	0.03
Other^‡^	92.0	90.1	0.07	92.9	92.3	0.02	96.1	96.4	0.02	97.6	98.4	0.06
Moved from outside of Ontario	—	83.5	—	—	86.8	—	—	94.1	—	—	96.8	—
No primary care visits	82.3	84.8	0.07	84.6	86.6	0.06	84.8	95.2	0.35	87.3	97.5	0.39
Material resources quintile												
Q1 (highest)	94.6	85.6	0.31	95.0	87.1	0.28	97.5	95.9	0.09	98.4	98.3	0.01
Q2	94.5	86.1	0.29	94.9	88.0	0.25	97.4	95.6	0.10	98.3	98.0	0.02
Q3	94.3	86.7	0.26	94.8	88.7	0.22	97.4	96.1	0.07	98.4	98.1	0.02
Q4	94.8	87.1	0.27	95.2	89.6	0.21	97.7	95.9	0.10	98.5	97.7	0.06
Q5 (lowest)	93.8	82.8	0.35	94.4	87.0	0.26	97.3	93.0	0.20	98.5	96.2	0.15

SD = standardized difference; UK = United Kingdom; USA = United States of America; UTD=up-to-date.

*Standardized differences (SDs) defined as the absolute difference in proportions in units of the pooled standard deviation compare immigrant or refugee children to Ontario-born children (35); SD > 0.1 were considered meaningful differences.

^†^Includes general practitioners, community medicine specialists, pediatricians and nurse practitioners who provide primary care in a family health team or through a comprehensive care model.

^‡^Includes walk-in clinics, solo-practitioners, and those with no primary care model.

**Fig 3:**
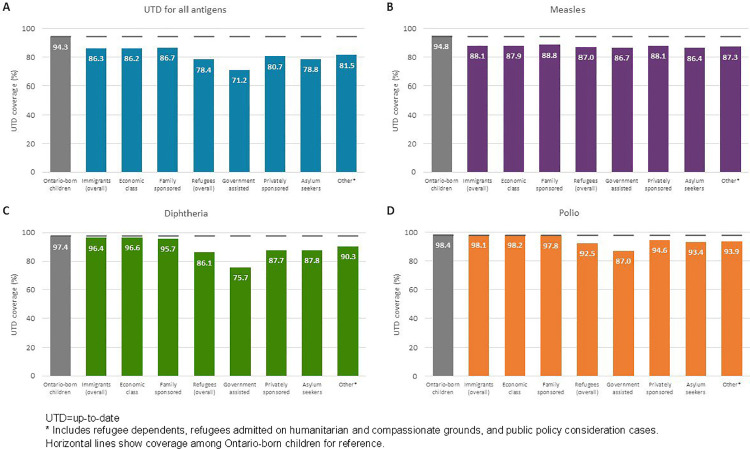
Up-to-date immunization coverage for (A) all antigens combined, (B) measles, (C) diphtheria, and (D) polio assessed at two years after school entry by immigration category and visa type among immigrant and refugee children in Ontario, Canada, 2014/15 to 2016/17 school years.

**Fig 4:**
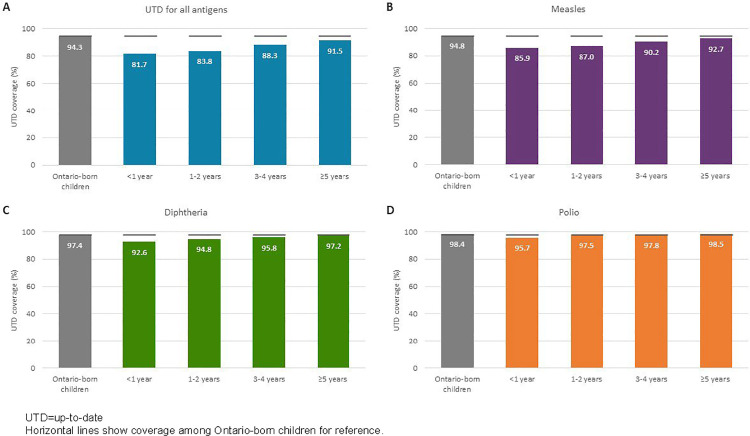
Up-to-date immunization coverage for (A) all antigens combined, (B) measles, (C) diphtheria, and (D) polio assessed at two years after school entry by immigration category and time since arrival among immigrant and refugee children in Ontario, Canada, 2014/15 to 2016/17 school years.

**Fig 5:**
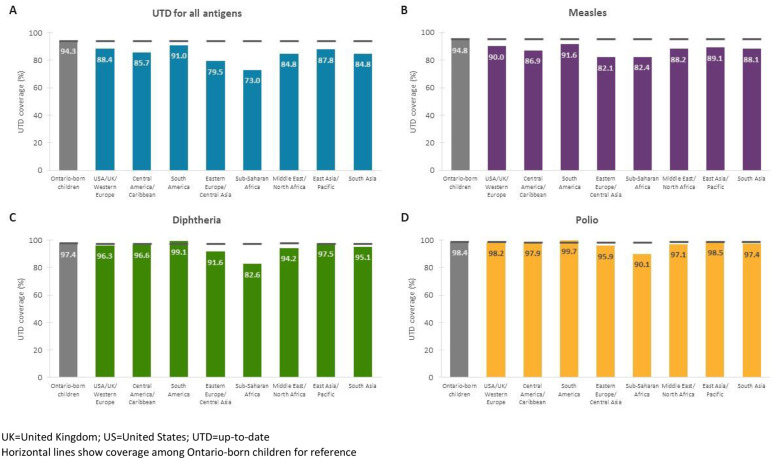
Up-to-date immunization coverage for (A) all antigens combined, (B) measles, (C) diphtheria, and (D) polio assessed at two years after school entry by region of birth among immigrant and refugee children in Ontario, Canada, 2014/15 to 2016/17 school years.

Among immigrant/refugee children, we found important variation in coverage based on immigration category, length of time in Ontario, and country of birth. Across all antigens, coverage was lower among refugee compared with immigrant children (Fig 3). Coverage was lowest among government-assisted refugees (Fig 3) and recent newcomers who landed in Canada less than five years ago (Fig 4). For diphtheria and polio, coverage was more similar between immigrant and Ontario-born children, with an increasing trend in coverage with a greater length of time in Ontario; for all antigens combined and measles, coverage also improved with more time spent in Ontario but still lagged behind Ontario-born children among those who had landed five or more years before school entry. We observed the largest absolute difference in coverage (>10%) in relation to country of birth. Coverage was lowest among immigrant/refugee children born in Eastern Europe, central Asia, and sub-Saharan Africa (Fig 5).

## Discussion

In a population-based cohort of children aged 5–6 years starting school for the first time in Ontario, we found that immigrant and refugee children had lower coverage of measles, diphtheria, and polio-containing vaccines relative to children born in Ontario hospitals. Coverage was lowest in refugees, recent immigrants, and those born in Eastern Europe, central Asia, and sub-Saharan Africa, consistent with prior research [3,7,12]. Coverage improved in this population after two years of school attendance and with increasing time spent in Ontario, but still lagged behind their Ontario-born peers, particularly for measles where coverage among immigrants/refugee children remained below herd immunity thresholds [15]. There are several reasons that might explain lower vaccine uptake among immigrant/refugee children, including language and cultural barriers, knowledge gaps, insufficient access to healthcare (either in Canada or their country of birth), trust in the healthcare system, and vaccine confidence [6,7]. Immigrant/refugee children are more likely to have missing or incomplete immunization records or be immunized according to different schedules or with different vaccine products that do not meet our provincial definitions for UTD coverage [5]. Also, because our provincial immunization repository relies on parental/guardian reporting, this lower coverage could be the result of underreporting as newcomer families are less familiar with legislative requirements or may have difficultly interpreting due to language barriers.

To our knowledge, ours is the first study to measure UTD immunization coverage of routine childhood vaccines among immigrant and refugee children in Ontario as distinct from children who were born in Canada to newcomer parents. Prior work using linked administrative databases in Ontario found higher UTD coverage for routine immunizations among 2-year-old children born to immigrant compared with non-immigrant parents [9]. Similarly, we previously found rotavirus vaccine uptake to be higher among children of recent immigrants in the initial years following program implementation in Ontario [10]. Immigrant parents tend to have lower vaccine hesitancy [37], and high levels of trust with healthcare providers and Canadian healthcare system [38,39]. They also have a lower likelihood of refusing, delaying, or being reluctant to receive vaccines, although these effects diminish with more years spent in Canada [40]. Together, this suggests that the lower coverage found in this study was more likely related to barriers to accessing immunizations, particularly among refugee children who experience disruptions to routine healthcare services due to war or conflict [41,42], and/or delays in reporting previously received immunizations to public health, rather than vaccine hesitancy or refusal. Indeed, we found that uptake improved with increasing lengths of time in Canada as newcomer families became more acculturated to the Canadian healthcare system, with immigrant/refugee children having the largest gains in coverage after two years of school attendance. Consistent with this hypothesis, a separate ICES-based analysis of COVID-19 vaccine uptake found higher single-dose coverage among immigrant children aged 4–10 years in Ontario compared with non-immigrant children, with lower coverage among resettled refugees and protected persons [12]. Expanded immunization program delivery during the COVID-19 pandemic (including through mass immunization clinics, mobile clinics, and pharmacists), immunization campaigns specifically targeted to racialized and ethnically diverse communities, and improved data collection methods (i.e., provincial COVID-19 vaccine registry) may explain this higher COVID-19 vaccine uptake among immigrant children during the pandemic [43].

We found that coverage was lowest for measles-containing vaccine, consistent with global trends of suboptimal 2-dose coverage (ranging from 33−91%) before the COVID-19 pandemic [44], potentially due to higher vaccine hesitancy for MMR vaccine [45]. This lower coverage might also be partially explained by the fact that we used a 2-dose UTD definition for measles coverage and did not consider doses given before 12 months of age as valid doses to align with NACI guidelines [34]. The number of countries offering a second dose of measles-containing vaccines has increased over time [46], with the World Health Organization only officially recommending a second dose since 2017 [47]. Several countries, including Bangladesh, India, Nigeria, Pakistan, and the Philippines, which were among the top 10 countries of origin in our study, did not introduce two-dose programs until 2009 or later [46]. Furthermore, in these countries and others where measles incidence and mortality remain high, the first measles-containing vaccine dose is often given as early as 9 months of age [47], which is not considered a valid dose under Canadian guidelines due to lower seroconversion rates for younger infants [34]. Unimmunized or under-immunized children immigrating to Canada are eligible for two doses of MMR/MMRV under Ontario’s publicly funded immunization schedule [14]. Given heightened measles activity worldwide in the post-COVID-19 pandemic era [48], targeted efforts will be required to fill in these gaps in coverage, particularly for recent immigrants and refugee children.

Routine infant and childhood immunizations in Ontario are almost exclusively administered through primary care providers. Immigration medical examinations, which are required for immigration to Canada, do not include a review of immunization status; instead, healthcare providers are advised to assess and update required immunizations for newcomers once they arrive in Canada [5]. Although resources have been developed to support clinicians who provide health services to newcomers [49,50], healthcare providers must balance these immunization assessments against other health concerns. In our study, coverage after two years of school attendance was highest among immigrant/refugee children receiving care from pediatricians, family physicians, or other primary care models and was lowest among those with no primary care visits. We also found that 2-year coverage was lower among immigrant/refugee children attending Community Health Centres, particularly for measles, compared with Ontario-born children. This finding was somewhat surprising since Community Health Centres are meant to deliver primary care services in high-needs areas to individuals who face barriers to care, including newcomers to Canada and those without health insurance, such as undocumented migrants [51]. These findings emphasize the importance of engagement in culturally-appropriate, patient-centred care for newcomer families [8,52]. Provider recommendation is one of the strongest predictors of vaccine uptake, particularly within immigrant communities [38], but can also often serve as a barrier for immigrants or refugees who may face language or cultural barriers without sufficient time or resources to address patients’ needs [6]. Our findings also underscore the important role that immigration officials, local schools, and public health units can play in ensuring newcomer families are made aware of what vaccines their children need, informing them how to get them, and ensuring the doses are received and documented.

Our study has several strengths, including a population-based immunization repository and the ability to link these immunization records to immigration data. However, there are some limitations to acknowledge. Immigrants and refugees represent a diverse population, including immigrants selected for their economic contributions or family reunification, refugees receiving government assistance or private sponsorship, asylum seekers, and undocumented individuals. Although we did not find differences in uptake by visa type, our analysis may mask heterogeneity across these sub-populations. Economic-class immigrants represented two-thirds of immigrant/refugee children in our study, which represent families with higher parental education, language ability, health literacy, and/or levels of skilled work experience. Our provincial immunization repository relies on parental/guardian reporting of immunizations delivered in primary care settings, which may affect its timeliness and completeness [25,26]. We were also missing data on several key covariates, including individual-level measures of household income, parental education, language proficiency, and measures of vaccine hesitancy. Finally, our study was conducted before the COVID-19 pandemic and some of the data are more than 10 years old. The disparities between newcomer and Canadian-born children have likely widened in the post-COVID-19 era due to disruptions to routine immunization programs during the pandemic [53–55], further increasing the risk of VPD outbreaks or imported cases, particularly for diseases such as measles and polio that have been eliminated in Canada [48,56]. Further research is needed to determine the impact of ongoing catch-up efforts during the pandemic-recovery period and any differential effects on immigrant or refugee communities.

Using linked health administrative data, we identified gaps in immunization coverage at school entry and after two years of school attendance among immigrant and refugee children in Ontario. Interventions to improve immunization coverage among newcomer children might include targeted community outreach, tailored education campaigns (including culturally-appropriate communications being made available in multiple languages), engagement with local community leaders, and removal of systemic barriers to access [7,8,52,57], along with incorporating lessons learned during the COVID-19 pandemic [12,58]. Ongoing efforts will be required to ensure that all Ontarians, including immigrant/refugee children, have timely and equitable access to routine immunizations and that Canada achieves its immunization coverage goals [15].
